# Can Insertion Sequences Proliferation Influence Genomic Plasticity? Comparative Analysis of *Acinetobacter baumannii* Sequence Type 78, a Persistent Clone in Italian Hospitals

**DOI:** 10.3389/fmicb.2019.02080

**Published:** 2019-09-12

**Authors:** Stefano Gaiarsa, Ibrahim Bitar, Francesco Comandatore, Marta Corbella, Aurora Piazza, Erika Scaltriti, Laura Villa, Umberto Postiglione, Piero Marone, Elisabetta Nucleo, Stefano Pongolini, Roberta Migliavacca, Davide Sassera

**Affiliations:** ^1^UOC Microbiologia e Virologia, Fondazione IRCCS Policlinico San Matteo, Pavia, Italy; ^2^Dipartimento di Scienze Clinico-Chirurgiche, Diagnostiche e Pediatriche, Università degli Studi di Pavia, Pavia, Italy; ^3^Pediatric Clinical Research Center, Dipartimento di Scienze Biomediche e Cliniche “Luigi Sacco”, Università degli Studi di Milano, Milan, Italy; ^4^Servizio di Epidemiologia Clinica e Biometria, Direzione Scientifica, Fondazione IRCCS Policlinico San Matteo, Pavia, Italy; ^5^Risk Analysis and Genomic Epidemiology Unit, Istituto Zooprofilattico Sperimentale della Lombardia e dell’Emilia Romagna, Parma, Italy; ^6^Dipartimento di Malattie Infettive, Parassitarie ed Immunomediate, Istituto Superiore di Sanitá, Rome, Italy; ^7^Dipartimento di Biologia e Biotecnologie “L. Spallanzani”, Università degli Studi di Pavia, Pavia, Italy

**Keywords:** *Acinetobacter baumannii*, genomic plasticity, ST78, insertion sequences, SMAL

## Abstract

*Acinetobacter baumannii* is a known opportunistic pathogen, dangerous for public health mostly due to its ability to rapidly acquire antibiotic-resistance traits. Its genome was described as characterized by remarkable plasticity, with a high frequency of homologous recombinations and proliferation of Insertion Sequences (IS). The SMAL pulsotype is an *A. baumannii* strain currently isolated only in Italy, characterized by a low incidence and a high persistence over the years. In this present work, we have conducted a comparative genomic analysis on this clone. The genome of 15 SMAL isolates was obtained and characterized in comparison with 24 other assemblies of evolutionary related isolates. The phylogeny highlighted the presence of a monophyletic clade (named ST78A), which includes the SMAL isolates. ST78A isolates have a low rate of homologous recombination and low gene content variability when compared to two related clades (ST78B and ST49) and to the most common *A. baumannii* variants worldwide (International Clones I and II). Remarkably, genomes in the ST78A clade present a high number of IS, including classes mostly absent in the other related genomes. Among these IS, one copy of IS66 was found to interrupt the gene *comEC/rec2*, involved in the acquisition of exogenous DNA. The genomic characterization of SMAL isolates shed light on the surprisingly low genomic plasticity and the high IS proliferation present in this strain. The interruption of the gene *comEC*/*rec2* by an IS in the SMAL genomes brought us to formulate an evolutionary hypothesis according to which the proliferation of IS is slowing the acquisition of exogenous DNA, thus limiting genome plasticity. Such genomic architecture could explain the epidemiological behavior of high persistence and low incidence of the clone and provides an interesting framework to compare ST78 with the highly epidemic International Clones, characterized by high genomic plasticity.

## Introduction

*Acinetobacter baumannii* is an opportunistic pathogen spread worldwide, which became clinically relevant in recent decades. Isolates of this species cause a wide range of nosocomial and community-acquired infections also due to their ability to colonize skin, mucous membranes, plastic intubation, and intravascular devices and is able to survive in the hospital environment (Peleg et al., 2008; Imperi et al., 2011). The genomes of thousands of *A. baumannii* isolates have been sequenced, showing a strong level of genome plasticity, in the form of a tendency to undergo frequent and substantial rearrangements, including recombinations (Snitkin et al., 2011; Feng et al., 2016) and movement of Insertion Sequences (IS) (Li et al., 2015).

Generally, the proliferation of IS elements in bacteria brings genomic variability, which can lead to adaptation to new niches; additionally high IS copy numbers have been linked to hypervirulence in multiple species (Rohmer et al., 2007; Beare et al., 2009). Moreover, IS elements act as anchors for homologous recombination processes, leading to internal genome rearrangements but also to the incorporation of exogenous DNA. Such homologous recombination events were found to be crucial for the evolution and adaptivity of *A. baumannii* and other pathogens; therefore, this genomic feature granted the bacterium a place among the so-called “bacterial hopeful monsters,” microorganisms able to rapidly modify their genotype through recombination events, and thus capable of quickly adapting to novel environmental conditions (Croucher and Klugman, 2014). Specific classes of ISs have been investigated in detail in *A. baumannii*. The presence of Insertion Sequence IS*Aba1* upstream the gene encoding for the beta-lactamase OXA-51 was suggested to grant *A. baumannii* resistance to carbapenem antibiotics (Turton et al., 2006). The gene *bla*_OXA–__51_ is always found in clinical isolates of *A. baumannii* (it is also known as oxaAb) (Nigro and Hall, 2016), while the presence of the IS might confer the resistant phenotype by boosting the expression of the beta-lactamase gene. In fact, several cases were reported of the IS being upstream of the resistance gene with the isolate remaining susceptible to carbapenems (Nigro and Hall, 2018). The expression enhancing activity of IS*Aba1* was also observed when associated with other genes, such as those belonging to the *bla*_ampC_ family (e.g., *bla*_ADC–__7_, *bla*_ADC–__52_), which drive resistance to Cephalosporins (Héritier et al., 2006).

The *A. baumannii* strains most commonly isolated in Europe belong to the two main International Clones, i.e., ICI and ICII (also known as European or Global Clones, EC or GC). In the early 2000s, these two clones were the first discovered to carry the plasmid-encoded gene *bla*_OXA–__58_, which confers resistance to carbapenems. More recently, strains of both International Clones have been reported to carry another determinant of resistance to carbapenems, the gene *bla*_OXA–__23_, which can be either encoded on plasmids or integrated into the chromosome (Turton et al., 2005). While reports of *bla*_OXA–__23_ strains are increasing, the gene *bla*_OXA–__58_ is being found less commonly in clinical isolates. This has been hypothesized to be due to the fitness cost that the gene carries (Migliavacca et al., 2013; Principe et al., 2014).

In the last 15 years, a different clone of *A. baumannii*, not evolutionary related to the two main International Clones, has been isolated multiple times in Italian hospitals. The clone was identified by Pulsed Field Gel Electrophoresis (PFGE) and named SMAL (based on the hospitals from which it was first isolated, San Matteo and Salvatore Maugeri acute care and long term care facilities, respectively) (Nucleo et al., 2009). Concurrently it was characterized by MultiLocus Sequence Typing (MLST, Pasteur scheme) as ST78, by multiplex-PCR as Sequence Group 6 (Giannouli et al., 2009, 2010), by rep-PCR as type 3 and by Amplification Fragment Length Polymorphism (AFLP) analysis as type 21, and finally deemed the “Italian Clone” (Carretto et al., 2011). Within a National survey on the spread of carbapenem-resistant *A. baumannii* strains, the majority of the isolates (52/55) in Italy belonged to ICII/ST2, while 3/55 genotyped strains showed a SMAL pulsotype (Principe et al., 2014). Despite the low number of isolates reported, the SMAL Clone represents an endemic reality in Italy since its first detection, in 2002.

SMAL strains often show resistance to carbapenems *in vitro* testing due mainly to the overexpression of the chromosome-encoded *bla*_OXA–__51_-like gene or the presence of resistance determinants (e.g., *bla*_OXA–__58_ and *bla*_OXA–__23_). The SMAL isolates collected earlier were characterized by the presence of a *bla*_OXA–__58_ determinant. This resistance gene was then found in 2009 to have been replaced in most isolates by a *bla*_OXA–__23_ gene (Nucleo et al., 2009; Carretto et al., 2011). Therefore, the SMAL Clone appears to have followed the same evolutionary pattern (in terms of acquisition and loss of resistance genes) of the International Clones I and II (Cherkaoui et al., 2015). Even SMAL isolates showing Minimum Inhibitory Concentration (MIC) values for carbapenems under the clinical susceptibility breakpoint can be very difficult to treat in case of localized and device associated infections, possibly due to a strong biofilm forming ability. This feature has been well demonstrated in previous studies, which showed how the biofilm production of SMAL isolates is considerably higher than that of the ATCC19606 reference strain (Nucleo et al., 2009; Carretto et al., 2011).

The aim of this work was to characterize the SMAL strains from a genomic point of view. Fifteen genome sequences were obtained and compared with evolutionary-related known ones. We focused our analyses mostly on gene content and genomic plasticity.

## Materials and Methods

### Availability of Data and Materials

The strains analyzed during the current study are available from the authors on request. Genome data generated are available in the EMBL EBI repository under the study accession number PRJEB19248 (EMBL-EBI), while plasmid sequences are deposited in NCBI GenBank as accession numbers KY202456, KY202457, KY202458, and KY652669 (NCBI-GenBank).

### Pulsed Field Gel Electrophoresis

PFGE of *A. baumannii* was performed after *Apa*I digestion using a method described previously (Bou et al., 2000). Genomic DNA was included in agarose plugs, and DNA restriction was carried out at 30°C for 16 h. PFGE was performed in a CHEF DRII system (Bio-Rad, Hercules, CA, United States), with pulses ranging from 0.5 to 15 s at a voltage of 6 V/cm at 14°C for 20 h. Lambda 48.5-kb concatemers (New England BioLabs, Beverly, MA, United States) were used as molecular size markers. Isolates showing three or fewer band differences were regarded as a single PFGE type, according to the criteria described previously by Tenover et al. (1995).

### Biofilm Formation Capability Assay

One milliliter of fresh medium in borosilicate (15 × 125 mm), polystyrene (12 × 75 mm) or polypropylene (12 × 75 mm) sterile tubes was inoculated with 0.01 ml of an overnight culture. Triplicate cultures for each sample were incubated for 8 h shaking (at 200 rpm in an orbital shaker) at 37°C. The supernatant of the tube was aspirated and rinsed thoroughly with distilled water. The cells attached to the tube walls were visualized and quantified by staining with crystal violet and solubilization with ethanol-acetone as described by Tomaras et al. (2003). The OD_600_ was determined using a spectrophotometer and compared to that of 2 MG and 65SM01 [i.e., known biofilm forming SMAL strains, already included in the work by Nucleo et al. (2009)].

### Identification and Antibiotic Resistance Profiling

Identification and susceptibility profiles were initially established using MicroScan4 (Beckman Colter) NBC46 panels. MICs of imipenem (IPM) and meropenem (MER) (carbapenem resistance) were obtained by Etest strips (bioMérieux). Results were interpreted according to the latest recommendations EUCAST guidelines (Eucast - Clinical Breakpoints, 2017).

### DNA Extraction, Sequencing, and Assembly

Bacterial strains were cultured in MacConkey agar medium. One single colony per strain was used for the downstream genomic analyses treated in this work. DNA was extracted using NucleoSpin Tissue (Macherey-Nagel) kit, libraries were prepared using Nextera XT kits and sequenced with the Illumina MiSeq technology with 2 × 250 paired-end runs. Reads were assembled with the Mira 4.0 assembler (Chevreux et al., 1999) using the default settings for Illumina reads and excluding the control for high coverage.

### Global Database of *Acinetobacter baumannii* Genomes

All available *A. baumannii* genomes (*n* = 1112, October 2017) were downloaded from the Patric website (PATRIC, the Pathosystems Resource Integration Center) using the search key “*Acinetobacter baumannii*.” Genomes sequenced in this work were added to the database. The Multilocus Sequence Type (MLST) of all genomes was determined using a previously published script (Gaiarsa et al., 2019) and the Pasteur profiling scheme (Diancourt et al., 2010). Genomes were filtered for quality following three criteria: a) genome size between 3.7 Mb and 4.3 Mb b) maximum number of contigs: 100 c) all MLST gene fragments are found complete using an automatic approach. If genomes sequenced in this work did not pass the filters, they were re-added to the dataset. Open reading frames (ORFs) were predicted in each genome using Prodigal (Hyatt et al., 2010). Single copy core genes were listed using BLAST by searching a previously published core genome (Higgins et al., 2017) against all gene sets, these were then aligned using Muscle (Edgar, 2004) and concatenated after removing potentially misaligned sites using Gblocks (Castresana, 2000). Phylogeny was performed using the software RAxML (Stamatakis, 2014) with the GTRGAMMA evolution model and 100 bootstrap replicates.

### Fine Phylogeny of the SMAL and Closely Related Strains

A concatenation of core genes detected by Roary (Page et al., 2015) was used as input for a phylogenetic analysis. The evolutionary model was predicted using ModelTest-ng (Posada and Crandall, 1998), while the phylogeny was performed using the software RAxML (Stamatakis, 2014) with the GTRGAMMAIX evolution model and 100 bootstrap replicates. A Bayesian molecular clock was performed with the software BEAST (Drummond and Rambaut, 2007), using as input the core genes concatenate and RAxML tree. Software parameters were set as in Gaiarsa et al. (2015): uncorrelated lognormal relaxed clock with the GTR model, with no correction for site rate heterogeneity. The analysis was run for 1,000,000,000 steps, discarded 100,000,000 steps as burn-in.

### Analysis of Recombination

In order to test for the presence of recombined regions, all 39 genomes analyzed were aligned to the evolutionary closest complete genome (which is strain 11510, according to the global phylogeny) using the Mauve software (Darling et al., 2004). Results were merged in a single multi-genome alignment which was used as input for ClonalFrameML (Didelot and Wilson, 2015) excluding all conserved sites. In detail, one preliminary run was performed (using 100 uncertainty simulations) in order to estimate the R/theta, 1/delta, and nu values posterior values (-emsim 100). Afterward, the analysis was repeated including the extracted starting values and the transitions vs. transversions mutation rate and with three different dispersion values (0.1, 0.5, and 1.0). Recombination rate was estimated by counting the total number of events and recombined bases in all terminal and intermediate nodes in each clade of interest. Values were normalized by dividing them by the number of nodes in the clade. In conclusion, the recombination rate of a clade was expressed as the average (of the three dispersion values) number of recombination events/node and as the average number of recombined bases/node.

### Pan/Core-Genome Analysis

All 39 genomes of the SMAL genomic neighborhood were annotated with the automatic pipeline Prokka (Seemann, 2014) using the default settings for bacteria, filtering paralogs and avoiding to call rRNA sequences. Orthologous groups were predicted using Roary (Page et al., 2015). An analysis of binary distance using gene presence as characters was performed using R. Dispersion in gene content for each clade of interest was calculated as follows: (pan-genome of the clade – core-genome of the clade)/number of organisms in the clade. The evolutionary distance between two genomes was expressed as the number of non-homoplasic core SNPs between the two genomes. Non-homoplasic sites were obtained using Noisy (Dress et al., 2008) on the core gene alignment previously used for the phylogeny of the ST78 genomic neighborhood. The evolutionary distance was plotted against the binary distance of gene content for each couple of genomes inside the clusters ST78A, ST78B, and ST49. Trendlines were calculated using the linear models and adding the R^2^ index (a statistical measure of how close the data are to the fitted regression line) in the R environment. Gene presence was re-tested using the mapping-based software SRST2 (Inouye et al., 2014) for confirmation. The pangenome previously obtained with Roary was used as a reference. Mapping reads for genomes TG22142, TG22146, TG22150, and UH1752 were sent to us by the respective authors. All other reads were available on the SRA database, except for those of the genomes 3909, UH5207, BM4578, and AB900, which were excluded from the analyses. A comparison of the gene plasticity of CC1, CC2, ST78A, and ST78B was performed with a similar method. The complete genomes of CC1 and CC2 were extracted from the global dataset of *A. baumannii* genomes. Gene presence was calculated using Prokka (Seemann, 2014) and Roary (Page et al., 2015) as described above. The evolutionary distance was calculated as described for ST78 by extracting non-homoplasic SNPs from the core gene alignment. In order to compare the trends in CC1, CC2 and the two clades of ST78, the evolutionary distance was normalized by dividing it by the total number of non-homoplasic sites in the respective core gene alignment. Moreover, only the evolutionary distances in the same range as those observed in the ST78 were used.

### Gene Content Analyses

The presence of genes coding for antimicrobial resistance and competence factors was tested using BLAST with an *ad hoc* prepared set of genes (see Supplementary Table S1). A database of virulence genes was obtained (Sahl et al., 2015) and tested with BLAST. More research was performed on the whole ResFinder and VirulenceFinder databases (CGE, 2017), using a permissive BLAST search and checking positive results manually. K-locus analysis was performed manually by BLAST-searching the regions between genes *fkpA* and *lldP*.

### Insertion Sequences Analysis

A collection of known ISs of *A. baumannii* was obtained from the ISSaga database (Varani et al., 2011; ISSaga, 2018) and was searched in all 39 genomes of the dataset using ISseeker (Adams et al., 2016). In order to confirm the result and to avoid assembly related biases, a mapping-based method was adopted. In detail, the available sequencing reads were mapped on the IS collection. The number of hits per sequence was normalized dividing by the length of each sequence. Inter-sample normalization was performed by dividing the levels of mapping reads by the average levels obtained by mapping the same reads on a collection of single copy core genes. Genes for normalization were selected starting from the result of the gene presence analysis, removing sequences that gave any hit when BLAST-searched against the IS collection.

Genes interrupted by different classes of ISs were detected by mapping reads against the IS collection and the pangenome. Paired ends in which one read mapped on the IS and the other one on the gene were marked as candidates. Strong candidates were defined as those with read pairs bridging the IS and the gene at both ends of the IS. Contingency of gene interruption with specific clades in the phylogeny was tested by calculating the φ (phi) coefficient and filtering all candidates with an absolute value score above 0.4.

### Plasmid Sequence Extraction and Characterization

Assembled genomes and contigs were BLAST-searched against an in-house generated database of plasmid replication sites of *A. baumannii*, while resistance genes were determined by uploading the contigs to ResFinder database (Zankari et al., 2012; ResFinder, 2017). Genomes containing a plasmid replication site were reassembled using SPAdes (Bankevich et al., 2012). Scaffolding was performed by manually inspecting the assembly graph using the software Bandage (Wick et al., 2015). Briefly contigs containing replicons and antibiotic resistance genes were linked to the adjacent contigs through the overlap sequences and graph links to obtain the final circular plasmids sequences. ORFs and their relative amino acids were predicted using Artemis (Rutherford et al., 2000). The annotation was performed manually using the online BLAST tool on the nr database.

## Results

### Genome Sequencing, Assembly, and Global Phylogeny

The total DNA of 15 isolates of *A. baumannii* previously recognized as SMAL was sequenced and assembled. One full carbapenamase encoding plasmid sequence was obtained from each of three genomes (see Tables 1, 2 for assembly data and accession numbers; one additional full plasmid not carrying a carbapenemase was also reconstructed in genome 5MO). The 15 SMAL isolates were assigned *in silico* to the ST78 and placed, on a global species phylogenomic tree, in a single monophylum together with 16 database genomes of the same ST (Supplementary Figure S1).

**TABLE 1:** Description of the 16 SMAL genomes, including the phenotypic characterization and the basic information on the assembled genomes and plasmids.

**Strain**	**City/Institute of isolation**	**MIC (μg/ml)**		**Biofilm formation capability (OD600)**			**Genome length (bp)**	**Genome N50**	**Plasmid accession number**	**Plasmid size (bp)**
							
		**MER**	**IPM**							
3909^∗^	Napoli, Ospedale Monaldi	32	6	0.4	0.4	0.4	3948828	31024	AEOZ01000236	26411
14336	Firenze, Ospedale Careggi	>32	>32	0.4	0.4	0.5	3961089	68038	KY202456	26496
103SM	Pavia, Policlinico San Matteo	1	0.38	0.6	0.7	0.5	3984084	119893		
20C15	Napoli, Ospedale Cardarelli	>32	>32	0.6	0.4	0.6	4014203	41098	KY202458	26781
25C30	Catania, Policlinico di Catania	>32	>32	0.5	0.5	0.5	4002419	63742		
2MG	Pavia, Fondazione Salvatore Maugeri	1.15	0.38	0.4	0.4	0.4	4028799	108463		
2RED09	Milano, Istituto Geriatrico “P. Redaelli”	>32	>32	0.4	0.4	0.6	3983268	119210	KY202457	25311
5MO	Monza, Ospedale San Gerardo	>32	>32	0.6	0.7	0.5	4026195	57707	KY652669	34233
61SM01	Pavia, Policlinico San Matteo	>32	0.38	0.5	0.5	0.3	4020527	129457		
65SM01	Pavia, Policlinico San Matteo	1.15	1.15	0.4	0.4	0.3	4002746	70283		
68SM01	Pavia, Policlinico San Matteo	>32	0.38	0.2	0.7	0.5	4015623	161914		
72SM01	Pavia, Policlinico San Matteo	0.5	0.38	0.6	0.7	0.8	4001559	61107		
74SM01	Pavia, Policlinico San Matteo	1.15	0.5	0.4	0.4	0.3	3969850	72068		
96SM	Pavia, Policlinico San Matteo	1	0.5	0.6	0.7	0.4	4010167	128042		
MGTN	Pavia, Fondazione Salvatore Maugeri	1	0.5	0.4	0.4	0.4	4002517	100890		
MONUR	Pavia, Fondazione Salvatore Maugeri	>32	0.38	0.5	0.5	0.5	4001892	88693		

*^∗^genome not presented in this work.*

**TABLE 2:** Feature summary of the 39 genomes compared in this work.

**Strain**	**Year of isolation**	**Country of isolation**	**Sequence Type/Clade**	**Carbapenem resistance gene**	**Isaba1 upstream *bla*_OXA–__51_ like**	**Isaba1 upstream *blaADC*_–__52_ like**	***bla*_CARB–PSE_**	***floR***	***sul2***	***aadB***	***aph (3′)-Ic***	***adeS***	***comEC/rec2***	**Accession number**
2MG	2012	Italy	ST78A (SMAL)			X	X	X	X	X	X	X	INT	GCA_900161905
25C30	2011	Italy	ST78A (SMAL)		X	X		X	X	X	X	X	INT	GCA_900161985
20C15	2011	Italy	ST78A (SMAL)	*bla*_OXA–__23_, *bla*_OXA–__58_		X		X	X	X	X	X	INT	GCA_900161955
3909	2007	Italy	ST78A (SMAL)	*bla*_OXA–__58_		X		X	X	X	X	X	INT	GCA_000189695
2RED09	2009	Italy	ST78A (SMAL)	*bla*_OXA–__58_		X		X	X	X	X	X	INT	GCA_900161995
14336	2010	Italy	ST78A (SMAL)	*bla*_OXA–__58_		X			X	X	X	X	INT	GCA_900161895
72SM01	2007	Italy	ST78A (SMAL)			X		X	X	X	X	X	INT	GCA_900162015
96SM	2012	Italy	ST78A (SMAL)			X		X	X	X	X	X	INT	GCA_900161925
103SM	2012	Italy	ST78A (SMAL)		X	X			X	X	X	X	INT	GCA_900161885
74SM01	2007	Italy	ST78A (SMAL)			X		X	X	X	X	X	INT	GCA_900161965
65SM01	2006	Italy	ST78A (SMAL)			X		X	X	X	X	X	INT	GCA_900161975
68SM01	2007	Italy	ST78A (SMAL)			X	X	X	X	X	X	X	INT	GCA_900161875
61SM01	2006	Italy	ST78A (SMAL)			X		X	X	X	X	X	INT	GCA_900161945
5MO	2009	Italy	ST78A (SMAL)	*bla*_OXA–__23_		X		X	X	X	X	X	INT	GCA_900161915
MGTN	2004	Italy	ST78A (SMAL)			X		X	X	X	X	X	INT	GCA_900161935
MONUR	2004	Italy	ST78A (SMAL)			X		X	X	X	X	X	INT	GCA_900162005
TG22142	2011	United States	ST78A		X	X	X	X	X	X	X	INT	X	GCA_000453965
TG22146	2011	United States	ST78A		X	X	X	X	X	X	X	INT	X	GCA_000453985
TG22150	2011	United States	ST78A		X	X	X	X			X	INT	X	GCA_000453025
ABUH393	2009	United States	ST78B	*bla*_OXA–__23_								X	X	GCA_002014105
ABBL026	2006	United States	ST78B									X	X	GCA_001432845
UH1752	2007	United States	ST78B									X	X	GCA_000809505
UH5207	2007	United States	ST78B									X	X	GCA_000516155
PR355	NA	United States	ST78B									X	X	GCA_002136855
1096934	2012	United States	ST78B									X	X	GCA_000581755
PR308	NA	United States	ST78B									X	X	GCA_002136675
PR385	NA	United States	ST78B									X	X	GCA_002138065
PR371	NA	United States	ST78B									X	X	GCA_002137185
855125	2012	United States	ST78B									X	X	GCA_000681555
831240	2012	United States	ST78B									X	X	GCA_000581975
ABBL025	2006	United States	ST78									X	X	GCA_001432665
BM4587	NA	United States	ST621									X	X	GCA_000731965
AB900	2003	United States	ST49									X	X	GCA_000173395
OIFC111	2003	United States	ST49									X	X	GCA_000309155
PR333	NA	United States	ST49									X	X	GCA_002137645
PR373	NA	United States	ST49									X	X	GCA_002137215
ARLG1941	NA	NA	ST49									X	X	GCA_002144835
PR389	NA	United States	ST49									X	X	GCA_002138155
1293320	2011	United States	ST49									X	X	GCA_000584455

*For Further information, see Table 1 and Supplementary Table S1. X, presence of a gene or phenotype; INT, interruption of a gene; NA, datum not available.*

### Refined Phylogeny

**FIGURE 1:**
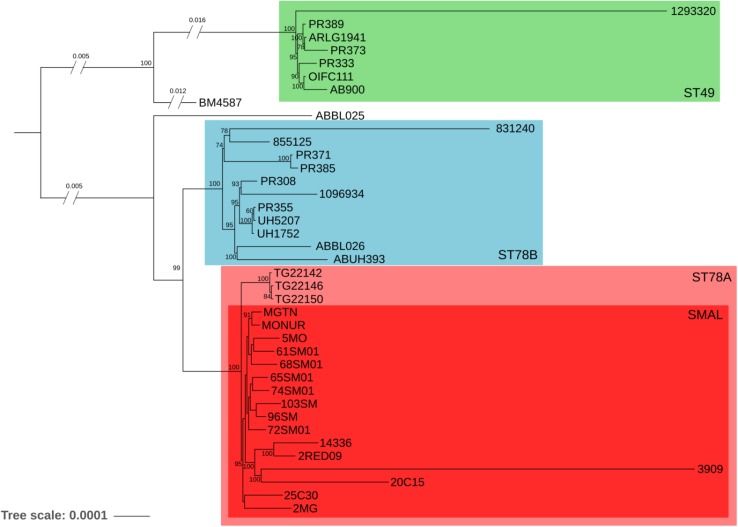
Maximum Likelihood phylogeny of 39 genomes, evolutionary related to the SMAL isolates obtained from a total of 2781 orthologous genes using the software RaxMLs with 100 bootstraps. The main clades are indicated with colored squares.

The phylogenomic surroundings of the generated genomes were inspected, and the ST78 monophylum, its sister clade (genome BM4587 with ST621) and the common sister group (composed of seven genomes of ST49, as observed in the global phylogeny) were subjected to in-depth analysis. Orthologous genes were predicted and the resulting core-genome of 2781 genes was used for a refined phylogeny which showed overall concordance with the global phylogeny (Figure 1). The ST78 monophylum was split into two well supported monophyletic groups (from now on called ST78A and ST78B). Within ST78A, the 15 novel SMAL genomes were clustered in a smaller single monophylum, together with strain 3909, previously isolated in Italy (Zarrilli et al., 2011). The isolate 3909 was obtained from the hospital of origin, analyzed with PFGE and attributed to the pulsotype SMAL as well. Within ST78A, three genomes isolated in Arizona (United States) in 2011 (J. Sahl, personal communication) form a highly supported clade that is positioned as sister group of the SMAL cluster. Recombination analysis was performed using multiple dispersion values with the software ClonalFrameML (Didelot and Wilson, 2015), clearly showing that ST78A genomes are less recombinogenic (average of 1.9 recombination events/node, 1100 recombined bases/node) than the genomes of the ST78B (average of 3.9 recombination events/node, 1859 recombined bases/node) and the ST49 clades (average of 5.3 recombination events/node, 9564 recombined bases/node). A Bayesian molecular clock analysis allowed to estimate the time of divergence of ST78A to 1980 (median = 31.7 years before 2012) and SMAL to 1985 (median = 27.1 years before 2012; Supplementary Figure S2).

### Phenotypic Characterization

Susceptibility profiles of the SMAL isolates were determined by *E*-test. Six out of 16 strains (3909, 2RED09, 14336, 5MO, 20C15, and 25C30) showed an intermediate/resistant phenotype to both Meropenem (MER) and Imipenem (IPM), with MIC values ranging from 6 to >32 mg/L (MIC50 ≥ 32 mg/L) (Table 1). Susceptibility was retained in 7/16 and 9/16 strains for MER and IPM, respectively. Full results of resistance profiles are reported in Table 1.

SMAL strains resulted to have, as expected, a strong biofilm formation ability, higher than that comparatively observed for the reference strain ATCC19606 (Nucleo et al., 2009; Supplementary Table S1). In detail, we show that all SMAL isolates have similar biofilm capability to 2MG and 65SM01, which were previously compared to the reference. Twelve Genes coding for biofilm formation were found in all the 39 genomes analyzed (SMAL and related ones), detecting multiple point mutations and two frame-shifting insertions (Supplementary Table S1). No particular pattern in relationship to the phylogeny was detected.

### Analysis of Gene Content

**FIGURE 2:**
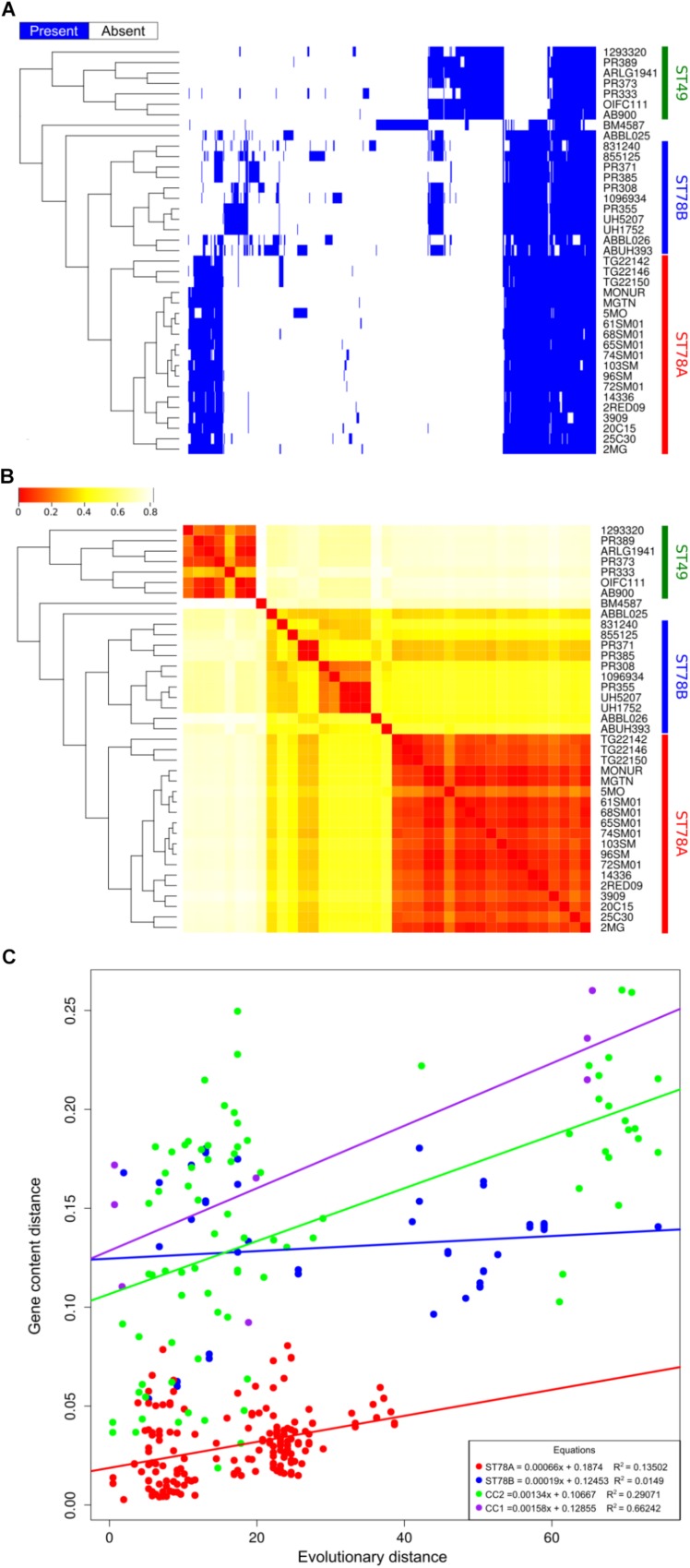
Genome plasticity of the 39 genomes in analysis. **(A)** Representation of the accessory genome content (blue areas represent the presence of the gene). **(B)** Heatmap representing the binary distance between all genome pairs. **(C)** Genome plasticity of ST78A (red), ST78B (blue), International clone I (ICI, purple), and International Clone II (ICII, green) represented by comparing the evolutionary distance and the gene content distance. Each dot on the scatterplot represents a pair of genomes. Gene content distance is calculated as the binary distance from an accessory gene presence matrix. Evolutionary distance is calculated as the number of non-homoplasic SNPs between two genomes, the distance is normalized on the total number of position in the alignment used for each clade.

Coding sequences were called and orthologous proteins were predicted in all the 39 genomes in the dataset. Results indicate that the gene content of the isolates of ST78A clade is highly conserved, with a gene dispersion rate of 35.58 genes/taxon (considering just SMAL genomes, the rate is 38.13 genes/taxon). The dispersion value is much higher in the ST78B clade (140.00 genes/taxon), while the ST49 clade scored 72.14 genes/taxon. The distribution of the accessory genes plotted on the tree shows the high similarity of the ST78A genomes (especially the SMAL cluster) compared to those of the ST78B and the ST49 clades (Figures 2A,B). This result prompted us to wonder whether the limited gene content variation of ST78A was indicative of low genomic plasticity, or just the consequence of smaller evolutionary distances. To address this question, we plotted the phylogenetic distances against distances of gene content for each genome pair within ST78A, ST78B and, for comparison, within International Clone I (ICI) and International Clone II (ICII) (Figure 2C). ICI and ICII genomes were chosen as representative of the average *A. baumannii* evolutionary behavior, as the two subclades comprise the two most commonly isolated strains in the clinical environment. The plot shows that the distance in gene content and the evolutionary distance present a direct correlation in all four genome groups. The intercept of ST78A is clearly lower than that of the other three groups, confirming the lower genomic plasticity observed in this clade.

We then investigated whether the gene content analysis could have been influenced by the assembly step, specifically due to different assembly pipelines used for the database genomes. Thus, we repeated the analysis with a different approach, by mapping the sequencing reads of each strain (where available) to a pangenome. Results confirm the highlighted trend of lower genomic plasticity in the ST78A clade (see Supplementary Figure S3).

### Resistome and Other Genes of Interest

Resistance gene content of the 39 genomes was analyzed by manually curated BLAST alignments against custom and public databases (Table 1 and Supplementary Table S1). IS*Aba1* is present in all the genomes of the ST78A cluster, while absent in most other genomes (with the exception of UH1752, ABUH393, and PR389). The sequence was found to be upstream the resistance gene *bla*_OXA–__90_ in five genomes (25C30, 103SM, TG22142, TG22146, TG22150). The carbapenem-resistance gene *bla*_OXA–__58_ was found in four SMAL genomes clustered in a highly supported monophylum (2RED09, 20C15, 14336, and 3909). The gene *bla*_OXA–__23_, instead, was detected in the genome of strains 5MO and 20C15. A *bla*_ADC–__52_-like gene was detected in all the 39 genomes in analysis. In all the 19 genomes of ST78A, a copy of IS*Aba1* was found 10 nt upstream of the gene. Furthermore, the *carO* gene (which encodes an outer membrane protein involved in carbapenem resistance) is present in all genomes; following the classification by Mussi and coworkers (Mussi et al., 2011), all genomes of ST78 have *carO* type I, while all ST49 genomes have *carO* type IV. The *adeS-adeR* two-component regulation system (involved in the fluoroquinolone resistance) are also found in all genomes. The sequence of *adeS* is interrupted by the end of the contig in the assemblies of the three strains from Arizona, suggesting the presence of an insertion sequence and a consequent loss of function.

Screening of virulence genes shows a very conserved distribution among the 39 genomes analyzed (Supplementary Table S2). A notable exception is the gene *atr2* (involved in the capsule biosynthesis), which is present only in the genomes of the ST78A clade. The presence of six competence-related genes was also tested. Notably, the gene *comEC/rec2* resulted to be interrupted in the assemblies of the 16 SMAL genomes, the interruption site always found at nucleotide 2146 (see Supplementary Table S1 for a full list of genes). Lastly, we characterized the K-locus of all 39 genomes analyzed. All ST78A resulted to have KL3, while all ST78B have a KL6A. ABBL025 has KL2, BM4587 has KL1 while all ST49 genomes have KL11.

### Analysis of the Mobilome

**FIGURE 3:**
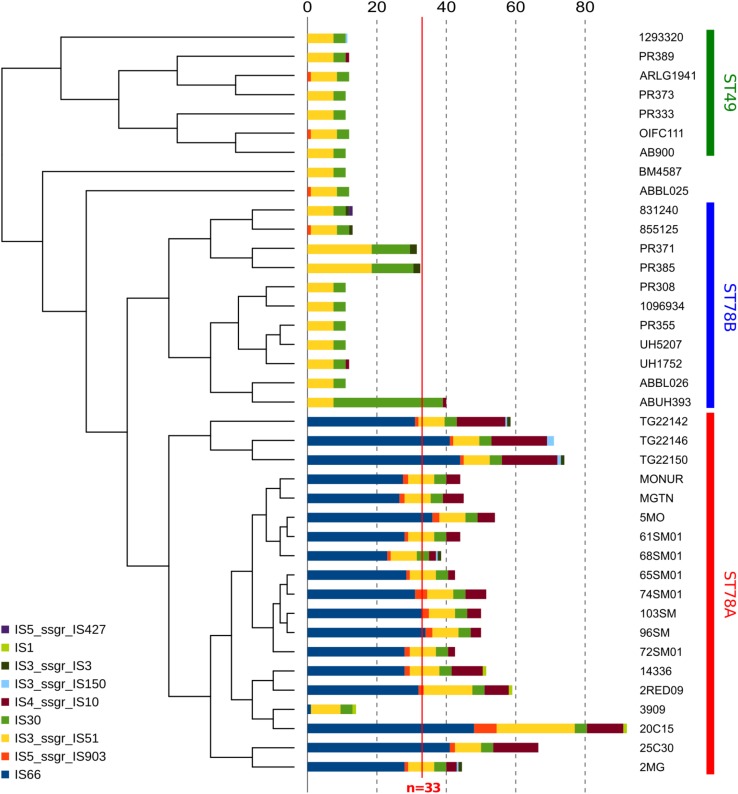
Abundance of different classes of Insertion Sequences (IS) in all 38 genomes in analysis. Data are plotted in histograms alongside the cladogram of the phylogeny (see Figure 1).

Insertion sequences (IS) analysis showed that the ST78A genomes contain strikingly more IS (average *n* = 52.26) than the other members of the clade (average *n* = 15.02), a much higher value than the average for the species (*n* = 33, in Adams et al. (2016); Figure 3 and Supplementary Table S2). This finding was confirmed using a mapping approach (Supplementary Figure S4 and Supplementary Table S3). This result prompted us to investigate whether the genes of interest that were found to be truncated in ST78A were interrupted by IS. The interruption in the competence gene *comEC/rec2* was found to be caused by an IS of the class IS66 within all the genomes of the SMAL clade. The gene *adeS*, interrupted in the three strains from Arizona, was also found to have an insertion of IS66 in all three cases. IS66 is a class that is rampant in ST78A genomes, while completely absent in the other genomes of the dataset. An in-house read mapping based approach (see description in the section “Materials and Methods”) was then used to identify all genes potentially inactivated by an IS insertion and the results were evaluated in terms of association of an insertion to a monophyletic group. The gene *comEC/rec2* resulted to be interrupted in all Italian genomes using this method as well. The interruption of two uncharacterized genes is a character statistically associated (φ > 0.8) with the ST78A clade and absent in the ST78B clade, while the interruption of 13 genes, including *adeS*, is statistically associated with the three genomes from Arizona in the ST78A cluster (φ > 0.6). Finally, the gene *yjcS* (coding for an alkyl sulfatase) is interrupted in most of the ST49 genomes but is never interrupted in the ST78 ones (φ = 0.54).

### Plasmid Analysis

Four SMAL genomes [including that of the previously sequenced strain 3909 (Zarrilli et al., 2011)] contain plasmids coding for *bla*_OXA–__58_ (Supplementary Table S1). The four plasmids have congruent size and good synteny (Supplementary Figure S5A), with some small internal reorganization. Conversely, the gene *bla*_OXA–__23_ was detected in strains 20C15 and 5MO and found to be chromosomally encoded. The *bla*_OXA–__23_ site was recognized in both cases to have a 100% sequence similarity with the previously described transposon *Tn2006* which was the first to be reported as a carrier of the resistance gene (Poirel et al., 2008). Part of the transposon sequence is shared with the genome of *Acinetobacter radioresistens* from which the gene was transferred to *A. baumannii* (Nigro and Hall, 2016). Plasmid annotation analysis showed a strong backbone similarity among the three newly described sequences, and with the one found in strain 3909 as well. All four sequences presented two *repA* variants (*repAci1* and *repAci2*) and two genes encoding for conjugal transfer proteins, *trbL* and *traA* (except for 3909 harboring only *traA*), followed by two IS*Aba25* insertion sequences. Some differences, however, were found around the *bla*_OXA–__58_ locus. Indeed, the plasmid of isolate 14336 (*pAB14336*) had opposite orientation of the IS*Aba2*/IS*Aba3-bla*_OXA–__58_-IS*Aba3* cluster when compared to the previously described plasmid *p183Eco* (Bertini et al., 2007). Moreover, one IS26 sequence is missing. On the plasmid of strain 2RED09, instead, *bla*_OXA–__58_ is surrounded by two IS*Aba3* with opposite orientation (Supplementary Figure S5B). On the plasmid of strain 20C15, *bla*_OXA–__58_ is flanked by IS*Aba3* and IS*Aba2*. Lastly, on the plasmid of 3909, *bla*_OXA–__58_ is flanked by IS*Aba2* and IS*Aba3* with different orientation for *bla*_OXA–__58_ when compared to p14336.

Two plasmid replication sites were found in the assembly of strain 5MO. One was the replication site of plasmid pAB5MO (published in the present work). The second was found on a contig possibly integrated into the chromosome. Indeed, the read coverage on the contig carrying the *repAci* (*aci6*) was similar to the average of the chromosome contigs. This plasmid was previously described as a carrier of carbapenemase genes (Bertini et al., 2010). As a matter of facts, the strain 5MO was found to encode a copy of gene *bla*_OXA–__23_ integrated into its chromosome.

## Discussion

The genomes of 15 strains of *A. baumannii* of SMAL pulsotype were sequenced and compared with the genomic variability of the species as a whole, and of closely related strains. A phylogenomic approach focused on 39 related genomes led to the identification of an evolutionary monophyletic group of 16 Italian genomes (estimated year of divergence: 1985) previously assigned to the SMAL pulsotype. Three other genomes isolated in the state of Arizona (United States) formed a closely related clade while the remaining 23 strains resulted more divergent. These data lead us to conclude that the SMAL clone is a monophyletic entity that was imported in Italy in one single event in the 1980s. Among SMAL genomes, that of strain 3909 stands out. In our results it is located on a long branch of the phylogeny (Figure 1) and it possesses a much lower number of IS copies than the other ST78A genomes (Figure 3). Indeed, 3909 is an old genome sequenced with 454 technology. This explains the long branch in the phylogeny, as 454 has higher error rate than Illumina. It could also explain the low number of IS, as low coverage and assembly with an older software, i.e., Newbler, has probably led to merging the repetitive regions. The unavailability of sequencing reads does not allow to test these hypotheses.

Four of the 15 genomes sequenced were found to carry plasmids. The complete sequence of the three plasmids carrying the gene *bla*_OXA–__58_ was compared with the ones in the databases, highlighting limited differences in the global structures of the novel plasmids. The site including the carbapenemase gene, on the other hand, showed high variability, especially concerning the IS. These variations surrounding the *bla*_OXA–__58_ locus suggest a lack of stability of this site. Such instability, together with the high energetic burden of maintaining a plasmid, could lead to events of loss of carbapenem resistance. These observations could indicate an ongoing switch, in epidemiological terms, from the plasmid-encoded *bla*_OXA–__58_ to the more stable and chromosome-mediated *bla*_OXA–__23_, found in two SMAL genomes. Other cases of replacement between the two determinants have been reported for other clones both in Italy and elsewhere (D’Arezzo et al., 2010; Wu et al., 2015). Furthermore, MIC values relative to carbapenems (Table 1) are mostly explained by the gene content (Supplementary Table S1). Five of the six resistant/intermediate isolates carry genes encoding for carbapenemases and the sixth (25C30) has the IS*Aba1* enhancer upstream the *bla*_OXA–__90_ gene. The isolate 103SM also presents the expression enhancing genotype but it has a carbapenem-susceptible phenotype. Such difference, especially in highly related isolates, may be an additional proof of upstream-IS*Aba1* not being sufficient to confer carbapenem resistance, as already reported by Nigro and Hall (2018). All four genomes carrying a plasmid encoded carbapenemase are monophyletic. This suggests that the plasmid could have been imported by the common ancestor of these strains, in one single event. Interestingly, two genomes with the same resistance gene profile showed completely different MIC values for meropenem. Unfortunately, gene content analysis did not allow to detect possible determinants of the high biofilm formation capability of the SMAL isolates. Variations were found in the genes responsible for biofilm biosynthesis (Supplementary Table S1) but none of them presented a pattern of presence/absence in the genomes that could suggest a relationship with the described phenotype.

The 16 Italian strains present three interesting genome features: (i) limited or absent recombination signal, (ii) highly conserved gene content (Figure 2), and (iii) a strong proliferation of multiple classes of IS elements, the richest being class 66 (Figure 3). These three characteristics seem to be in contrast, as the first two features suggest genome stability, while IS proliferation is considered a trademark of genomic plasticity. Here we propose a genome evolution scenario that starts with the proliferation of ISs, including IS66 elements. One IS66 then inactivated the gene *comEC/rec2*, an event clearly shown by our genome data to have occurred just once, at the basis of the Italian clade. ComEC is an inner membrane protein responsible for the intake of DNA in the cytoplasm (Krüger and Stingl, 2011; Wilharm et al., 2013). In the proposed scenario the interruption of this gene could have reduced the capability of DNA exchange of the Italian strains. In parallel, IS elements proliferation played other roles in the evolution of the clade, such as affecting the stability of the *bla*_OXA–__58_ locus, not found in the most recent genomes, and causing the loss of function of other genes, which could have in turn contributed to the current low genomic plasticity of the entire ST78A clade, or to branches of it. Furthermore, we observed that the genomic plasticity of ST78A is lower also when compared to the most commonly isolated variants of *A. baumannii*, i.e., the International Clone I and International Clone II. These key elements suggest that the force driving the evolution of the SMAL clone could be IS elements, acting as selfish DNA (i.e., genetic segments that can spread throughout the genome or transfer to other individuals, regardless of the effect on the fitness of the organism). IS are known to be, usually, anchors for homologous recombination processes and are thus considered carriers of genomic plasticity and responsible for the evolution of virulent clones in multiple bacterial species. In the case described in the present work, the SMAL clone possesses a high number of IS elements but surprisingly there is evidence of limited homologous recombination. On the contrary, the interruption of the *comEC/rec2* gene by IS66 may contribute to the reduction of import of exogenous DNA. Thus, in this case, recombination events do not seem to be coupled with IS proliferation but appear to be in a competitive relationship.

## Data Availability

The strains analyzed during this study are available from RM on reasonable request (roberta.migliavacca@unipv.it). The genomes generated for this study can be found in the EMBL EBI repository (https://www.ebi.ac.uk/ena/data/view/PRJEB19248). The plasmid sequences generated for this study can be found in the NCBI GenBank with the accession numbers KY202456, KY202457, KY202458, and KY652669 (https://www.ncbi.nlm.nih.gov/nuccore/).

## Ethics Statement

Neither ethics committee approval, nor informed consent were required as all collected data were fully anonymized, there was no contact with patients and/or their families and no interventions or changes to treatment and management were made, in accordance with local guidelines.

## Author Contributions

SG, RM, PM, and DS designed the study. MC and PM performed the microbiological analysis. IB, AP, and EN performed the molecular biology analysis. ES and SP performed the genome sequencing. IB and LV performed the plasmid bioinformatic analysis. SG, UP, and FC performed the genome bioinformatic analysis. SG and DS drafted and finalized the manuscript. IB and RM contributed to the writing of the manuscript. All authors read, corrected, and approved the manuscript.

## Conflict of Interest Statement

The authors declare that the research was conducted in the absence of any commercial or financial relationships that could be construed as a potential conflict of interest.

## Supplementary Material

FIGURE S1 Global phylogeny of *Acinetobacter baumannii*. Phylogeny of 1112 genomes of *A. baumannii*, representing the evolution of the species. SMAL sequences are highlighted in red, while the 39 genomes further characterized in the study are highlighted in blue; International Clone I is indicated in green, International Clone II is indicated in red; the tree is represented as a cladogram with no branch lengths for better visualization.

FIGURE S2 Bayesian molecular clock of the 39 genomes in analysis. Molecular clock analysis of 39 strains. Time is measured in years before the most recent strain isolation date (i.e., 2012).

FIGURE S3 Genome plasticity of the 39 genomes in analysis, calculated by read mapping. **(A)** Heatmap representing the accessory genome content (blue areas represent the presence of the gene). **(B)** Heatmap representing the binary distance between all genomes. No read data was available for genomes 3909, UH1752, AB900, and BM4587.

FIGURE S4 Insertion sequences detected by read mapping. Abundance of different classes of Insertion Sequences (IS) in 35 genomes as calculated by mapping reads to the IS sequences (no read data was available for genomes 3909, UH1752, AB900, and BM4587). Data are plotted in histograms alongside the cladogram of the phylogeny.

FIGURE S5 Genomic structure of the four plasmids carrying the resistance gene *bla*_OXA–58_. **(A)** global synteny as calculated with the Mauve software. **(B)** Topology of the resistance gene locus, after manual annotation.

TABLE S1 Gene content of the 39 strains in analysis. Results of the refined analysis of presence of genes of interest of the following categories: A. Resistance. B. Virulence. C. Competence. D. Biofilm formation. Indicated mutations are calculated in comparison with the genome of strain 2MG (first column).

TABLE S2 Insertion sequences detected with ISseeker. Number of Insertion Sequences (ISs) detected by ISseeker per each class in all genomes in analysis.

TABLE S3 Insertion sequences detected by read mapping. Coverage of Insertion Sequences (ISs) per each IS class, calculated by reads mapping on 35 genomes (no read data was available for genomes 3909, UH1752, AB900, and BM4587).
