# Supplementary figures and images for: Can Insertion Sequences Proliferation Influence Genomic Plasticity? Comparative Analysis of Acinetobacter baumannii Sequence Type 78, a Persistent Clone in Italian Hospitals
Source: Front Microbiol. 2019 Sep 12;10:2080. doi: 10.3389/fmicb.2019.02080 (PMC6751323; doi:10.3389/fmicb.2019.02080)

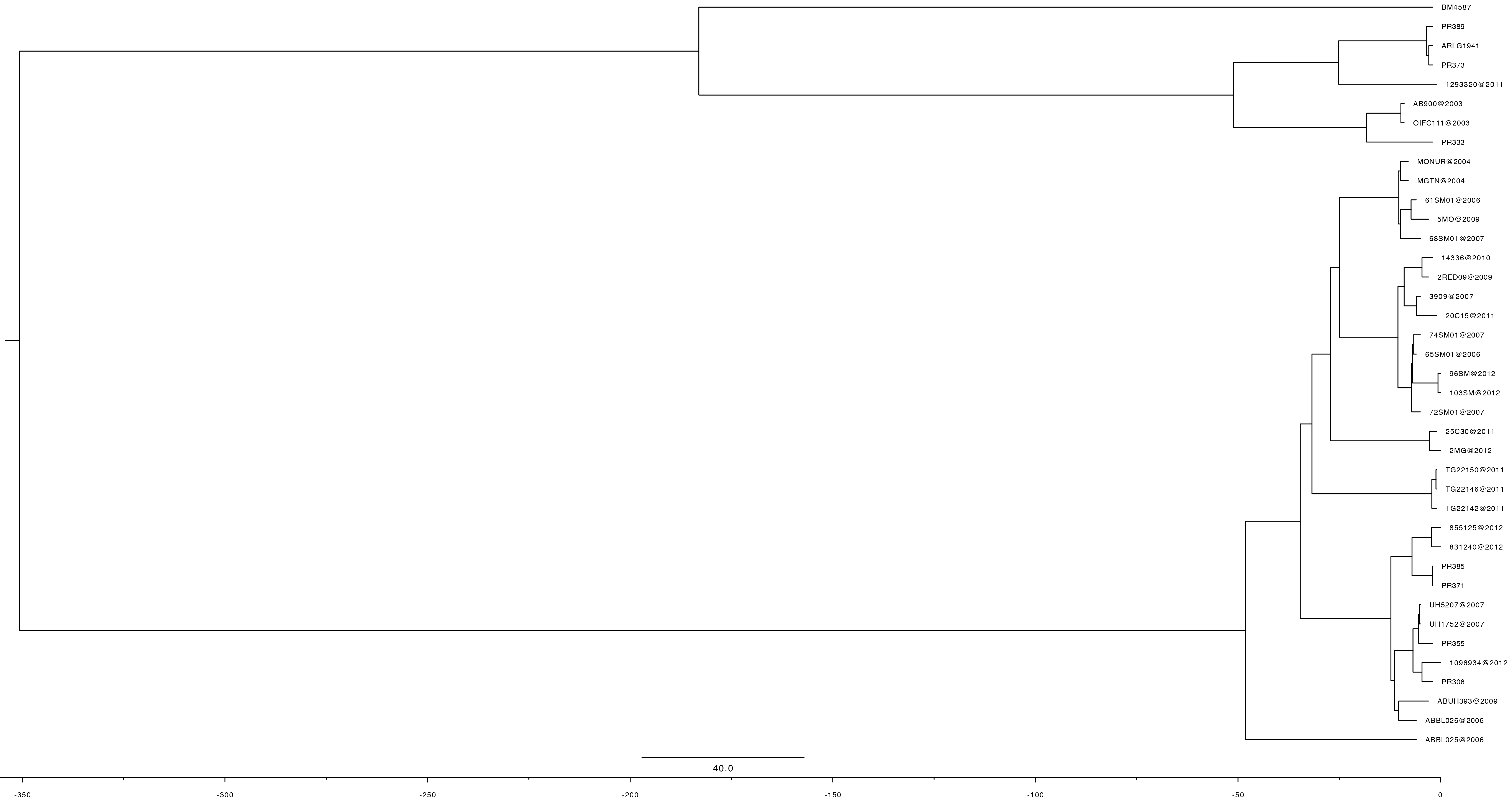

Supplement: FIGURE S2 — Bayesian molecular clock of the 39 genomes in analysis. Molecular clock analysis of 39 strains. Time is measured in years before the most recent strain isolation date (i.e., 2012). [file Image_2.TIF]

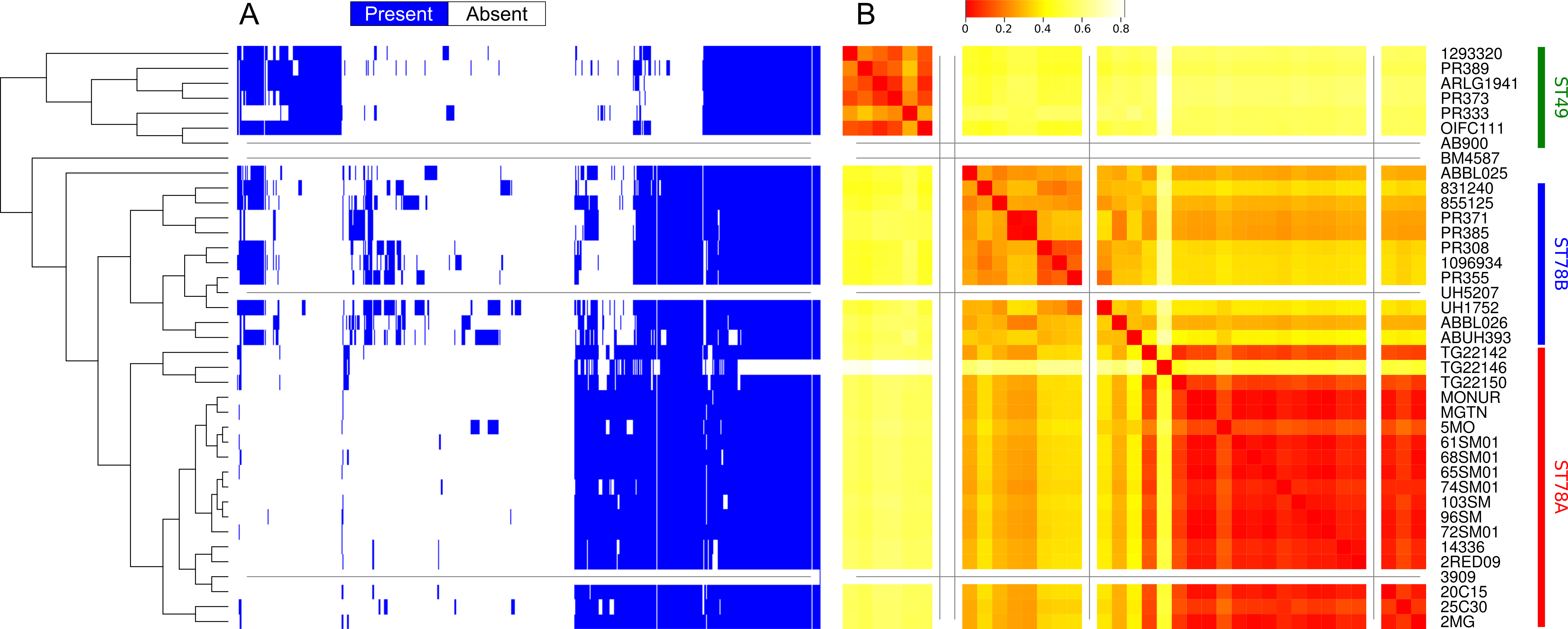

Supplement: FIGURE S3 — Genome plasticity of the 39 genomes in analysis, calculated by read mapping. (A) Heatmap representing the accessory genome content (blue areas represent the presence of the gene). (B) Heatmap representing the binary distance between all genomes. No read data was available for genomes 3909, UH1752, AB900, and BM4587. [file Image_3.PNG]

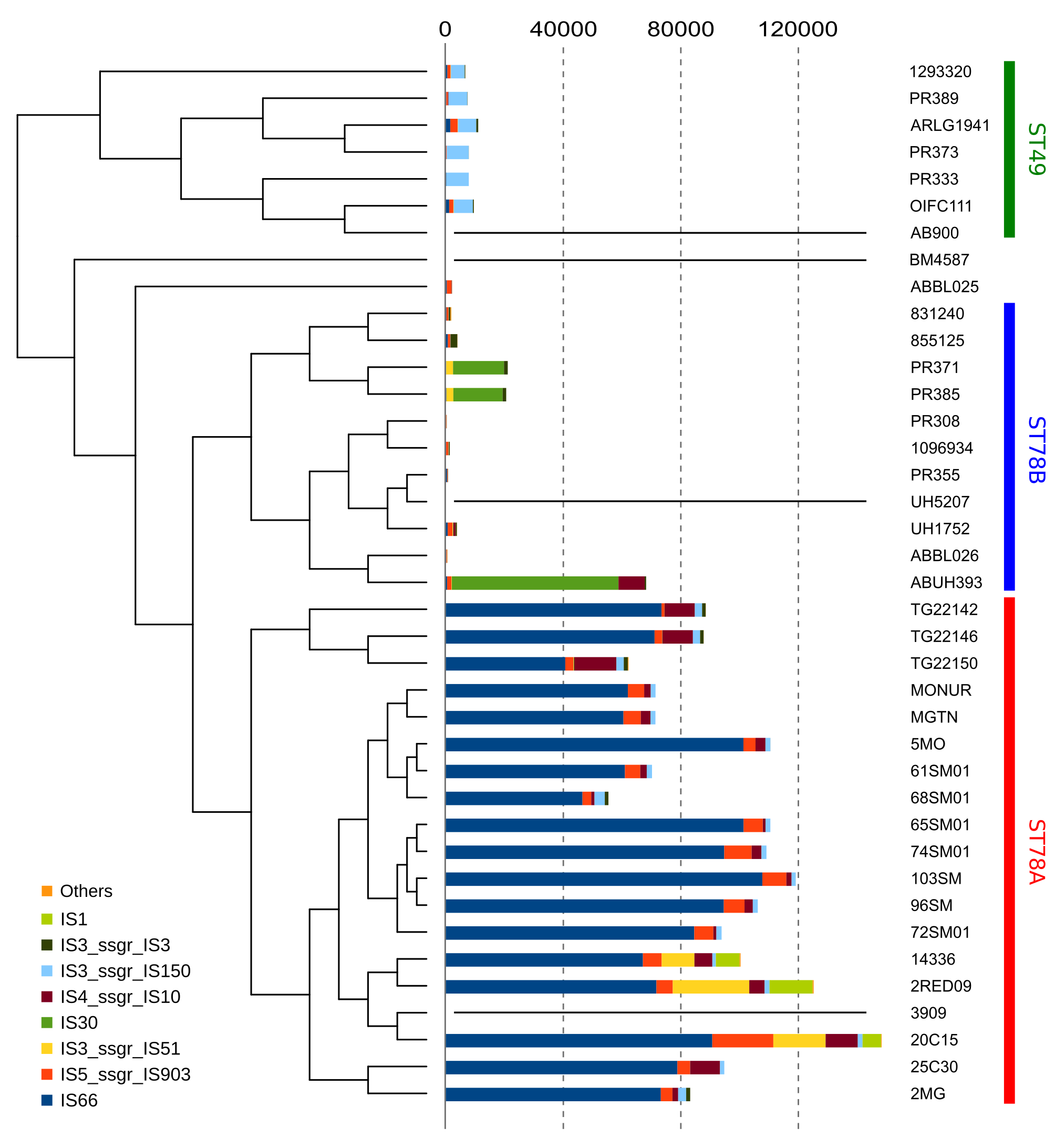

Supplement: FIGURE S4 — Insertion sequences detected by read mapping. Abundance of different classes of Insertion Sequences (IS) in 35 genomes as calculated by mapping reads to the IS sequences (no read data was available for genomes 3909, UH1752, AB900, and BM4587). Data are plotted in histograms alongside the cladogram of the phylogeny. [file Image_4.PNG]

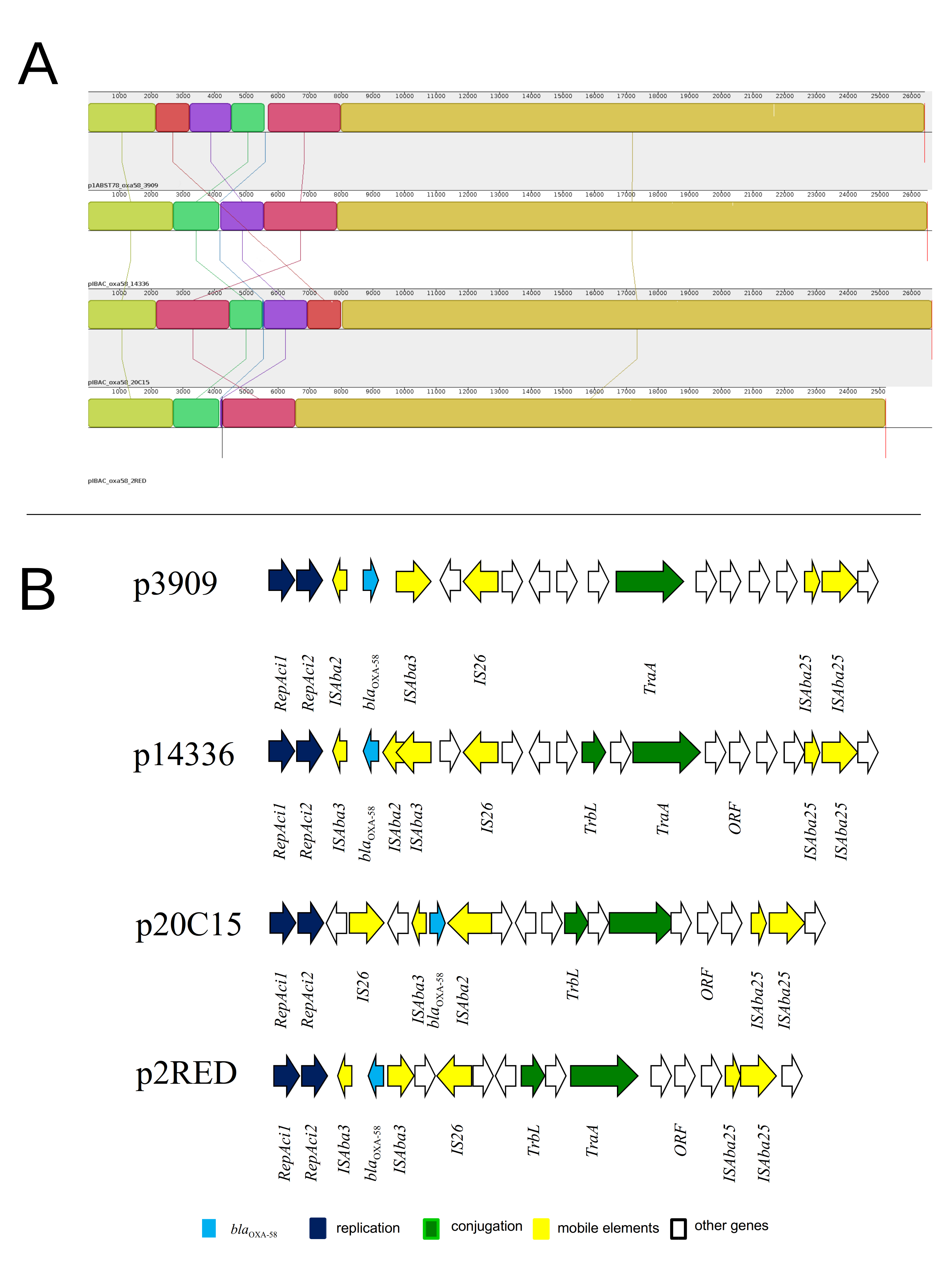

Supplement: FIGURE S5 — Genomic structure of the four plasmids carrying the resistance gene blaOXA–58. (A) global synteny as calculated with the Mauve software. (B) Topology of the resistance gene locus, after manual annotation. [file Image_5.PNG]
